# Anticancer Effects of the Marine Sponge* Lipastrotethya* sp. Extract on Wild-Type and p53 Knockout HCT116 Cells

**DOI:** 10.1155/2017/7174858

**Published:** 2017-01-03

**Authors:** Kiheon Choi, Hyun Kyung Lim, Sung Ryong Oh, Woo-Hyun Chung, Joohee Jung

**Affiliations:** ^1^College of Pharmacy, Duksung Women's University, Seoul 01369, Republic of Korea; ^2^Innovative Drug Center, Duksung Women's University, Seoul 01369, Republic of Korea

## Abstract

Interest in marine bioresources is increasing in the drug development sector. In particular, marine sponges produce a wide range of unique metabolites that enable them to survive in challenging environments, which makes them attractive sources of candidate pharmaceuticals. In previous study, we investigated over 40 marine specimens collected in Micronesia and provided by the Korean Institute of Ocean Science and Technology, for their antiproliferative effects on various cancer cell lines, and* Lipastrotethya* sp. extract (LSSE) was found to have a marked antiproliferative effect. In the present study, we investigated the mechanism responsible for its anticancer effect on wild-type p53 (WT) or p53 knockout (KO) HCT116 cells. LSSE inhibited cell viability and induced apoptotic cell death more so in HCT116 p53 KO cells than the WT. HCT116 WT cells treated with LSSE underwent apoptosis associated with the induction of p53 and its target genes. On the other hand, in HCT116 p53 KO cells, LSSE reduced mTOR and Bcl-2 and increased Beclin-1 and LC3-II protein levels, suggesting autophagy induction. These results indicate that the mechanisms responsible for the anticancer effect of LSSE depend on p53 status.

## 1. Introduction

Primitive marine animals produce a diverse range of metabolites that enable them to flourish in the marine environment, and in particular, the wealth of unique metabolites produced by marine sponges has attracted the attention of those trying to develop new drugs, including anticancer drugs. Several articles have been published on the biological activities of marine sponges [1, 2], but the mechanisms responsible have yet to be elucidated.

Generally, agents that induce apoptosis or autophagy are of interest as potential cancer therapies [3, 4]. Apoptosis is called programmed cell death [5], is a critical developmental process, and is essential for tissue remodeling. Cells undergoing apoptosis exhibit characteristic features, such as DNA fragmentation and cytoplasm shrinkage, and because cancer cell apoptosis causes tumor regression, it is utilized for chemotherapy [6].

**Figure 1:**
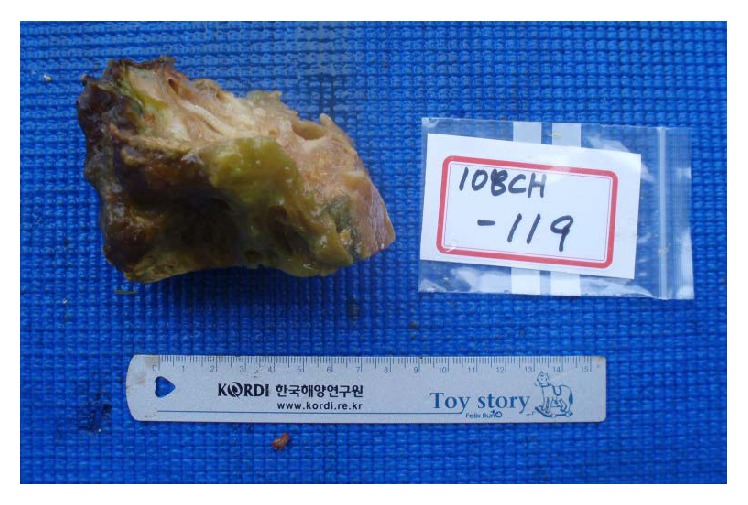
Morphology of* Lipastrotethya* sp.

Autophagy controls cellular homeostasis and survival [7]. Initially, autophagy was regarded a tumor-suppressive mechanism, but it is also related to tumor progression [7, 8]. These conflicting effects of autophagy depend on cancer type and the microenvironment [9]. Thus, the cell death induced by autophagy could conceivably be utilized for anticancer therapy. On the other hand, p53 had an important anticancer effect and is functionally associated with apoptosis, autophagy, genome stability, and the cell cycle [10–12]. In a previous study, we found that the marine sponge* Lipastrotethya* sp. (Figure 1) inhibited the proliferation of cancer cells. In the present study, we investigated the mechanism responsible for its anticancer effects in HCT116 WT and HCT116 p53 KO cells.

## 2. Materials and Methods

### 2.1. Specimen Preparation

*Lipastrotethya* sp. extract was kindly provided by H.-S. Lee (Korea Institute of Ocean Science and Technology). The sample was collected by scuba diving in Chuuk state, Federated States of Micronesia, washed with sterilized artificial sea water three times, immediately frozen, and stored at −20°C until required. The lyophilized specimen was extracted with methanol and dichloromethane; then it was dissolved in sterile distilled water. Aliquots of the sample were stored at −20°C until required [13, 14].

### 2.2. Cell Culture

Human colorectal carcinoma HCT116 (expressed wild-type p53) and HCT116p53KO cells were cultured in Dulbecco's modified Eagle's medium (DMEM, GenDEPOT) supplemented with 10% fetal bovine serum (GenDEPOT) and 1% penicillin/streptomycin (GenDEPOT) in a humidified 5% CO_2_ incubator. Cells used for assays were in the exponential growth phase.

### 2.3. Cytotoxicity

Cell cytotoxicities were determined using the Cell Counting Kit-8 (CCK-8, DOJINDO, Japan) as previously described [14]. Briefly, cells were seeded in 96-well plates at 3 × 10^3^ cells/well, incubated for 24 h, and treated with LSSE for 48 h. CCK-8 reagent (10 *μ*L) was added to each well and incubated for 3 h at 37°C. Absorbance at 450 nm was determined using a microplate reader (Infinite M200 PRO, TECAN, Austria).

### 2.4. Western Blot

Cells were seeded in a 6-well plate at 4~6 × 10^4^ cells/well. Samples were treated, added to each well, and incubated for 24 h. Cells were harvested and lysed in RIPA buffer (GenDEPOT) containing protease inhibitors (Xpert protease inhibitor cocktail solution, GenDEPOT) and phosphatase inhibitors (Xpert phosphatase inhibitor cocktail solution, GenDEPOT). Cell lysates were boiled in 5x sample buffer and separated by 10% SDS-PAGE. Proteins were transferred to PVDF membranes (Millipore) using a semidry electro blotter (Peqlab, Germany). Membranes were blocked with 5% skim milk in TBST (50 mM Tris-HCl pH 7.4, 150 mM NaCl, 0.1% Tween 20) and incubated sequentially with primary antibodies at 4°C, overnight. Membranes were then incubated for 1 h at room temperature and probed with secondary antibody. Immunoreactive proteins were visualized using ECL reagents and detected using Chemi-Doc. Antibodies and the used were p53 (1 : 2000, Upstate), Hdm2/MDM2 (1 : 1000, Bioss), p14/Arf (1 : 1000, Cell Signaling), p21 (1 : 2000, Millipore), *β*-actin (1 : 5000, Sigma-Aldrich), Bax (1 : 1000, Cell Signaling), caspase-9 (1 : 1000, Cell Signaling), caspase-3 (1 : 1000, Cell Signaling), cleavage caspase-3 (1 : 1000, Cell Signaling), mTOR (1 : 10,000, Abcam), PUMA (1 : 1000), NOXA (1 : 1000), Bcl-2 (1 : 1000), Beclin-1 (1 : 1000, Cell Signaling), LC3 (1 : 2000, Abcam), anti-mouse IgG (H + L) horseradish peroxidase conjugate, and anti-rabbit IgG (H + L) horseradish peroxidase conjugate (1 : 3000, Bio-Rad).

### 2.5. Apoptosis Assay

HCT116 and HCT116 p53KO cells were seeded in six-well plates and treated with LSSE for 24 h. Incubated cells were stained using the Annexin V-FLUOS staining kit (Roche, Mannheim, Germany). Apoptotic cells were counted under an optical microscope (×20, DMi8, Leica, Germany).

### 2.6. Statistical Analysis

Results are presented as means ± standard deviations. Student's *t*-test was used to determine the significance of intergroup comparisons, and statistical significance was accepted for *p* value < 0.05.

## 3. Results

### 3.1. Inhibition of Cell Viability by* Lipastrotethya* sp. Extract

**Figure 2:**
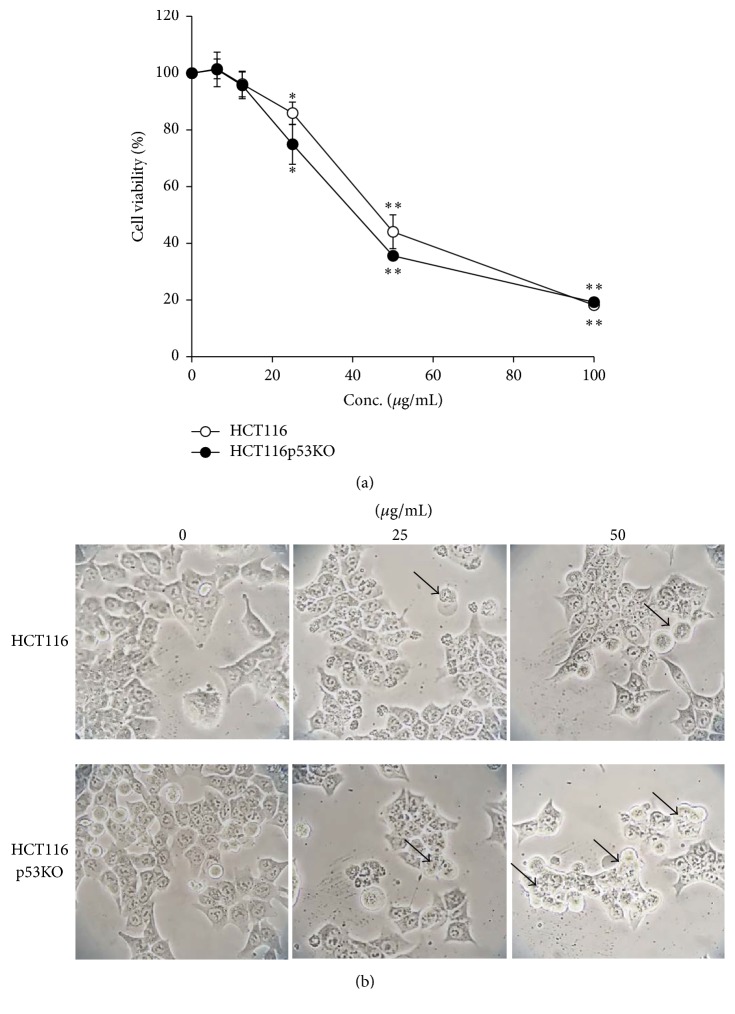
Inhibition of cell viability by* Lipastrotethya* sp. extract. (a) Cells treated with* Lipastrotethya* sp. extract were incubated for 48 h. Cell viability was determined by Cell Counting Kit-8 as described in Materials and Methods. Data showed mean ± standard deviation (*n* = 8). ^*∗∗*^*t*-test (*p* < 0.01). (b) Morphology of cells treated with* Lipastrotethya* sp. extract. Cells were incubated for 24 h and observed by microscope (×40). ^*∗*^*t*-test (*p* < 0.05).

HCT116 and HCT116 p53KO cells were treated with* Lipastrotethya* sp. extract (LSSE) for 48 h and cell viability was determined using Cell Counting Kit-8. LSSE dose-dependently reduced cell viability in both cell lines (Figure 2(a)). The IC_50_ of LSSE was 44.8 and 38 *μ*g/mL in HCT116 and HCT116 p53KO cells, respectively. LSSE inhibited the viability of HCT116 p53KO cells more so than that of HCT116 cells. Furthermore, HCT116 and HCT116 p53KO cell numbers were decreased and their morphologies were changed after the treatment with LSSE for 24 h (Figure 2(b)). In particular, HCT116 p53KO cells treated with LSSE (50 *μ*g/mL) were spherical and floated (black arrows) on culture medium. These results suggested that HCT116 p53KO cells were more sensitive to LSSE than HCT116 cells.

### 3.2. *Lipastrotethya* sp. Extract-Induced Apoptosis

**Figure 3:**
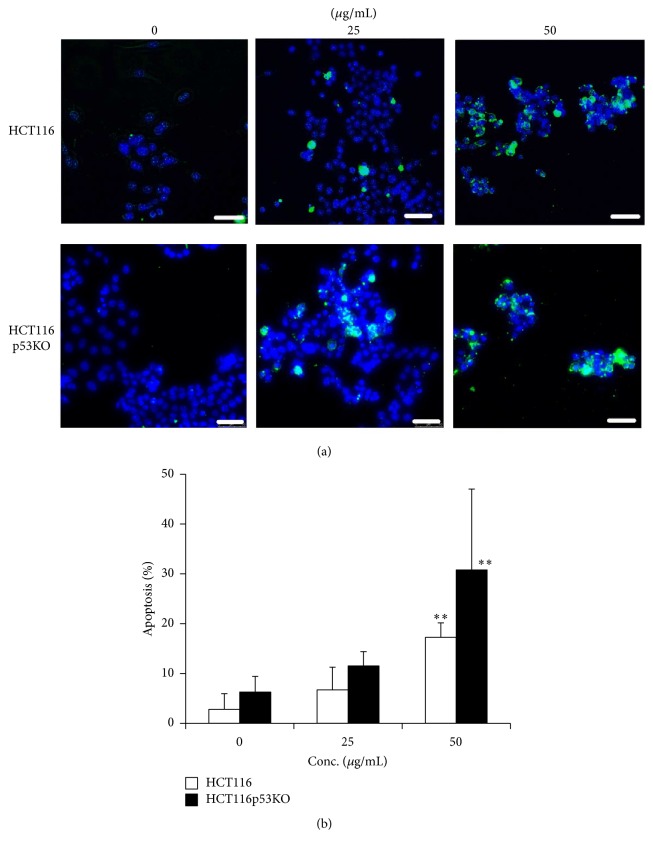
Induction of apoptosis by* Lipastrotethya* sp. extract. Cells treated with* Lipastrotethya *sp. extract were incubated for 24 h. Apoptotic cells were measured by Annexin V staining as described in Materials and Methods. (a) Green fluorescent cells show apoptotic cells. DAPI staining represents nucleus (Blue). Scale bar, 50 *μ*m. (b) Data showed mean ± standard deviation (*n* = 5). ^*∗∗*^*t*-test (*p* < 0.01).

To elucidate the mechanism underlying the cell death induced by LSSE, we measured apoptotic cell numbers using an Annexin V-FITC assay. As shown in Figure 3(a), Annexin V-positive (apoptotic) cells were dose-dependently increased by LSSE in both cell lines. Percentages of apoptotic cells to total cells showed LSSE (50 *μ*g/mL) significantly induced early apoptosis in HCT116 cells (17.2%) and HCT116 p53KO cells (30.8%) (Figure 3(b)). These results indicate LSSE induced apoptosis in both cell lines (Figure 2). Furthermore, LSSE induced more apoptosis in HCT116 p53KO cells.

### 3.3. Different Anticancer Mechanism of* Lipastrotethya* sp. Extract on HCT116 and HCT116p53KO Cells

**Figure 4:**
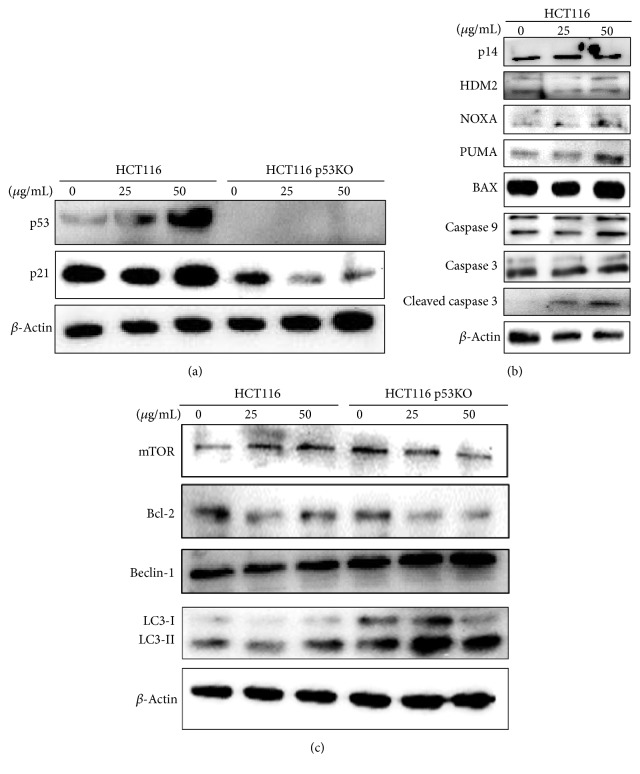
Effect of* Lipastrotethya* sp. extract on the expression of cell death-related proteins. Cells treated with* Lipastrotethya* sp. extract were incubated for 24 h. (a) p53 level and p21 level in HCT116 and HCT116 p53KO cells treated with* Lipastrotethya* sp. extract. (b) Apoptosis-related protein expression was analyzed by western blotting in HCT116 cells treated with* Lipastrotethya* sp. extract. (c) Autophagy-related protein expression was analyzed by western blotting in HCT116 and HCT116 p53KO cells treated with* Lipastrotethya* sp. extract.

P53 is key player in apoptotic cell death. Above all, p53 expression was confirmed in two cell lines (Figure 4(a)). As was expected in HCT116 WT cells, LSSE dose-dependently increased p53 levels and increased the levels of the p53 targeted genes p21 (Figure 4(a)) as well as NOXA, PUMA, and Bax (Figure 4(b)). Accordingly, we investigated the expressions of caspase 9 and caspase 3. Cleaved caspase 9 and cleaved caspase 3 were slightly increased by the treatment of LSSE (Figure 4(b)). These results suggested that LSSE caused p53-mediated apoptosis through the intrinsic pathway in HCT116 cells. Additionally, we investigated the expression of p14/Arf and HDM2 as p53 regulators in HCT116 treated with LSSE (Figure 4(a)). The levels of p14 and HDM2 showed no difference between control and LSSE treatment. LSSE might induce p53 induction via the other pathway.

On the other hand, p53 deficiency was confirmed in HCT116 p53KO cells regardless of LSSE (Figure 4(a)). P21 as one of p53-targeted genes is known to be a cyclin-dependent kinase inhibitor and arrests cell cycle [15]. Also, p21 can cause apoptosis via the p53-independent pathway [16]. Thus, we investigated p21 levels in HCT116 p53 KO cells after treatment with LSSE, but no induction of p21 was observed (Figure 4(a)). Nevertheless, because LSSE had a greater apoptotic effect in HCT116 p53 KO cells, we further investigated the mechanism of cell death in these cells. As shown in Figure 4(c),* Lipastrotethya* sp. suppressed mTOR and Bcl-2 levels and increased LC3-II and Beclin-1 levels. As these factors are markers of autophagy, there results suggested that LSSE induced autophagy-related cell death in HCT116 p53KO cells.

## 4. Discussion

The present study was undertaken to investigate the mechanism responsible for the anticancer effect of the extract of* Lipastrotethya* sp. (LSSE) on wide-type p53 (WT) and p53 knockout (KO) HCT116 cells. LSSE had an antiproliferative effect on HCT116 cells regardless of p53 expression, and interestingly, the IC_50_ of LSSE in HCT116 p53KO cells was lower than in HCT116 cells (Figure 1). Usually, induction of p53 expression triggers apoptosis and increases anticancer effects because p53 is a tumor-suppressor gene. In the present study, LSSE increased the expressions of p53-target genes like p21, PUMA, and NOXA and induced apoptosis* via* intrinsic pathway in p53 expressing WT HCT116 cells (Figure 4(b)). Furthermore, we investigated whether LSSE activates apoptosis via the Arf-HDM2/Mdm2-p53 tumor-suppressor pathway. P14/Arf is known to control the p53 activation* via* suppression of HDM2 [17]. However, our results showed no difference of p14 level between control and LSSE treatment as well as HDM2. In particular, HDM2 acts as both positive and negative regulator of p53 activity depending on stresses [18]. These results suggested that LSSE might induce the p53 level in response not to oncogenes stress but to DNA damage. Generally, deletion or loss of p53 is associated with chemoresistance and a poor prognosis [19]. Furthermore, cancer cells exhibiting p53 deletion or loss are aggressive and associated with poor survival [20]. Thus, new anticancer drugs are needed to treat aggressive or resistant cancers exhibiting p53 deletion or loss. In this study, LSSE triggered autophagy in HCT116 p53 KO cells by decreasing mTOR and increasing LC3-II protein levels (Figure 4(c)) and, thus, induced cell death (Figure 3). These results suggested that LSSE is good candidate treatment for p53-deficient colorectal cancer cells. Unfortunately, the active components of LSSE responsible for cell death mediated autophagy in p53 KO cells were not identified. Nevertheless, several components such as nortriterpene glycosides of sarasinoside have been previously isolated from* Lipastrotethya* sp. and shown to be toxic to A549 cells [21]. In addition, triterpene galactosides of pouoside have also been identified in* Lipastrotethya* sp. [22]. Several triterpenoids isolated from marine animals have been shown to have anticancer activities [23] and this structure has the benefits for several disease including cancers [24]. Moreover, our findings regarding the anticancer effects of* Lipastrotethya* sp. concur with those of previous studies.

## 5. Conclusions

Our results indicate that the mechanisms responsible for the anticancer effect of LSSE depend on p53 status: that is, in the HCT116 WT cells LSSE induced apoptosis via the intrinsic pathway but in HCT116 p53 KO cells, LSSE induced an autophagic response.
